# Emergency Endoscopic Interventions in Acute Upper Gastrointestinal Bleeding: A Cohort Study

**DOI:** 10.3390/diagnostics13233584

**Published:** 2023-12-01

**Authors:** Anna Mackiewicz-Pracka, Piotr Nehring, Adam Przybyłkowski

**Affiliations:** Department of Gastroenterology and Internal Medicine, Medical University of Warsaw, 02-091 Warsaw, Poland; anna.mackiewicz@wum.edu.pl (A.M.-P.); piotr.nehring@wum.edu.pl (P.N.)

**Keywords:** acute, bleeding, gastrointestinal, endoscopy, therapy, upper, varices

## Abstract

Introduction: Acute upper gastrointestinal bleeding is a common cause of emergency department admissions. The standard approach for the diagnosis and treatment of acute upper gastrointestinal bleeding (AUGIB) involves an endoscopy of the upper gastrointestinal tract. While daytime emergency endoscopy has been well studied, there is limited evidence regarding its effectiveness during the nighttime. Patients and Methods: We conducted a retrospective cohort study at a single center, analyzing adult patients with AUGIB referred for emergency endoscopy outside of regular hospital hours. Patients treated with endoscopic hemostatic methods were categorized into day-hours and night-hours groups based on the timing of the gastroscopy. The primary clinical endpoint was 120-day all-cause mortality, with secondary endpoints including hemostasis and recurrence. Results: In the population of 752 enrolled patients with acute upper gastrointestinal bleeding symptoms, 592 had a gastroscopy during the day hours between 8.00 *a.m.* and 10.00 *p.m.*, while 160 had procedures performed at night between 10:00 *p.m.* and 8:00 *a.m.* In the day-hours group, the median time from symptom onset to endoscopy was 10 h (IQR 6–15), compared to 6 h (IQR 4–16) in the night-hours group. The gastroscopy duration (time to reach hemostasis during endoscopy) was significantly shorter during the night hours (*p* < 0.001). In both groups, endoscopic intervention after the sixth hour from symptom onset yielded improved outcomes, while treatment before the fifth hour resulted in poorer outcomes. Although the night-hours group had higher 120-day all-cause mortality, the difference was not statistically significant. Conclusions: Our findings indicate that emergency therapeutic gastroscopy for acute upper gastrointestinal bleeding is similarly effective during both day and night hours, particularly when performed after the sixth hour from symptom onset.

## 1. Introduction

Patients with symptoms of acute upper gastrointestinal bleeding (AUGIB) are admitted to the emergency department at least once every week. The incidence of AUGIB is estimated to be approximately 80 to 150 per 100,000 people per year [1]. The effective management of AUGIB significantly impacts patient outcomes. Various medical societies have issued guidelines, each with their distinctive recommendations. According to all guidelines, endoscopy is the diagnostic and therapeutic standard in patients with AUGIB symptoms. In line with the European Society of Gastrointestinal Endoscopy (ESGE) guidelines, gastroscopy with endoscopic treatment, if necessary, should be performed within 24 h from presentation to the hospital [2]. It is important to properly prepare the patient for a planned endoscopic procedure. It is necessary to conduct an assessment of their vital signs, as well as the collection of a detailed history regarding the circumstances, time of occurrence, duration of symptoms, comorbidities, and medications. Additionally, laboratory tests should be performed, such as blood morphology, coagulation tests, biochemical tests, and blood group. If necessary, packed red blood cells and fresh frozen plasma should be ordered for eventual transfusion. Hemodynamic resuscitation in patients with AUGIB involves supplementing the vascular bed with crystalloids, which aims to restore proper tissue perfusion, thus preventing multi-organ failure and reducing mortality. Decisions on pharmacotherapy depend on the suspected etiology of bleeding: variceal [3] or non-variceal [4,5,6,7,8]. In the case of non-variceal bleeding, the patient should be treated with a proton pump inhibitor, while in variceal bleeding, following the latest Baveno Consensus from 2022, vasoactive drugs (terlipressin, somatostatin, octreotide) should be started as soon as possible and continued for 2–5 days [3]. Proton pump inhibitor (PPI) administration is a common recommendation in all guidelines. It serves as an adjunct to endoscopy, effectively reducing re-bleeding, especially in patients with high-risk lesions [2,9]. Hospital admission and the monitoring of AUGIB patients are endorsed in all guidelines. The ESGE, in particular, recommends a minimum of 72 h of observation following successful endoscopic therapy [2,9]. Regarding transfusion thresholds, the Japanese guidelines offer specific recommendations favoring restrictive transfusion strategies [10]. The management of anticoagulants and antiplatelet agents should adhere to the guidelines of the relevant medical societies, such as the American Society for Gastrointestinal Endoscopy (ASGE) and the American Gastroenterological Association (AGA) [9]. The guidelines uniformly underscore the importance of the diagnosis and eradication of *H. pylori* infection, especially in patients with peptic ulcers [2,9].

An important step in the management of patients with symptoms of AUGIB is assessment and risk stratification before proceeding with endoscopy [11]. For this purpose, medical professionals employ the Glasgow–Blatchford score, which assesses the risk of bleeding from the gastrointestinal tract before initiating an endoscopic examination and helps to determine the likelihood of the need for endoscopic intervention [12]. Furthermore, the Rockall score is utilized to assess the patient both before and after endoscopy [13,14]. The assessment of a patient with AUGIB using an appropriate predictive scale ensures the possibility of selecting the optimal treatment method [15]. Emphasis on risk stratification is consistent across all recommendations. The ASGE, the AGA, the Asia–Pacific working group, the Korean Society of Gastroenterology, and the Japan Gastroenterological Endoscopy Society (JGES) recommend the use of the Rockall or Glasgow–Blatchford scores to identify high-risk patients [9,16,17]. The ESGE also echoes the significance of risk stratification [2].

Endoscopic hemostasis is uniformly advised during the initial endoscopy for patients with active bleeding and displaying high-risk stigmata of bleeding, including visible vessels, adherent clots, or ulcers with exposed vessels, according to all guidelines [2,9]. This has led to the imposition of a 24 h endoscopic duty in hospitals. There is an ongoing discussion regarding the optimization of endoscopy time, focusing on the criteria for patients’ qualification for urgent endoscopy (<6 h), early endoscopy (6–24 h), and deferred endoscopic intervention (>24 h). Early endoscopy enjoys the unanimous endorsement of these guidelines. The ASGE advocates upper endoscopy within 24 h of admission [9]. The ESGE, the Asia–Pacific working group, and the Korean Society of Gastroenterology similarly support the early implementation of endoscopy [2,16,17]. The time to endoscopy can be shortened to less than 12 h in high-risk patients, including those who are hemodynamically unstable despite drug–fluid resuscitation. There is no clear definition of a high-risk patient; therefore, an individual approach and clinical assessment is recommended.

Second-look endoscopy is encouraged by the ASGE and the AGA, typically performed within 12–24 h after endoscopic therapy to confirm hemostasis and guide further management [9]. According to the ESGE and the Korean Society of Gastroenterology, second-look endoscopy is not recommended due to the high risk of re-bleeding. However, it may be considered when there are symptoms of re-bleeding, ineffective or unreliable endoscopic hemostasis, or in the case of a high suspicion of re-bleeding, e.g., unstable vital signs [16,18]. In cases where endoscopic therapy proves ineffective hemostasis or in instances of persistent or recurrent bleeding, the consideration of early surgical intervention is suggested, with the timing determined on an individual basis as per ASGE and AGA guidelines [9].

Hospitals with endoscopic facilities maintain a round-the-clock endoscopic duty staffed by qualified medical personnel. The primary objective of such organized healthcare is to ensure the most effective and patient-centric intervention in the event of symptoms of acute upper gastrointestinal bleeding. This comprehensive approach involves coordinating the endoscopic team (doctor and nurse), the anesthesiology team (doctor and nurse), access to an equipped endoscopy room, and the availability of the necessary equipment and tools. Additionally, there should be a post-operative room or ward or, in more severe cases, access to an intensive care unit for continuous medical care following endoscopy. It is essential to note that any endoscopic intervention not preceded by optimal management, incorrect qualification for therapeutic endoscopy, or improper timing can result in additional costs and the potential need for repeat endoscopy. Therefore, ongoing efforts are being made to optimize the availability of on-call endoscopic procedures and identify factors influencing their effectiveness. This retrospective study aims to provide a characterization of the emergency endoscopic treatment of AUGIB performed during round-the-clock duty, with a special focus on the efficacy and safety related to the time of day when these gastroscopies are carried out.

## 2. Material and Methods

### 2.1. Patients

The analyzed population included all consecutive patients of the University Clinical Center of the Medical University of Warsaw, Poland, who were treated with endoscopic intervention due to AUGIB in the period from July 2016 to December 2020 outside the normal working hours of the hospital. This study is a retrospective analysis of available medical records.

The inclusion criterion for the study was an endoscopic intervention for acute upper gastrointestinal bleeding undertaken outside of normal working hours, i.e., between 3:00 *p.m.* and 8:00 *a.m.* on weekdays and 24 h a day on non-working days. The exclusion criteria were the absence of AUGIB symptoms, a confirmed bleeding site in the middle or lower gastrointestinal tract, and endoscopic intervention performed during regular office hours (Monday to Friday between 8.00 *a.m.* and 3.00 *p.m.*). We opted to omit this interval from the study since our hospital boasts a team of at least five proficient endoscopists available to collaborate during regular working hours each day. In contrast, only one endoscopist is accessible during specific duty hours, mirroring the conditions in smaller hospitals. Medical records were analyzed in terms of basic demographic data, the cause of bleeding (variceal or non-variceal), the timing of endoscopic intervention, and the effectiveness of the endoscopic therapy. The Glasgow–Blatchford score and ‘pre-endoscopy’ Rockall score values were analyzed if data were available to calculate them. For this study’s purpose, the patients were divided into two groups: therapeutic gastroscopies performed during the day hours (between 8.00 *a.m.* and 10.00 *p.m.*) and in the night hours (between 10.00 *p.m.* and 8.00 *a.m.* and on days off work, including bank holidays). Therapeutic success was defined as the identification of the site of active bleeding during upper gastrointestinal endoscopy and hemostasis achieved during the index endoscopy, plus no need for repeated endoscopic therapy or surgery. Therapeutic success rate, 120-day all-cause mortality, and repeated therapeutic endoscopy rate were compared in both groups.

This study was approved by the Ethics Committee at the Medical University of Warsaw, Poland. The present study is retrospective and is based on medical history and patient records, without any interventions or possible risks to patients; thus, informed consent was not sought.

### 2.2. Statistical Analysis

A statistical analysis was performed with Statistica data analysis software system (version 13; Dell Inc., Tulsa, OK, USA) and PQStat ver. 1.6.6.204 (PQStat Software, Poznań, Poland). A Student’s *t*-test was performed for parametric variables; a Mann–Whitney *U* test was used for nonparametric quantitative variables. Parametric and nonparametric variables were recognized based on their distribution. The χ^2^ test was used with Fisher’s correction for small samples in a qualitative variables analysis. Missing data were removed in pairs. Statistical significance was recognized when *p* < 0.05.

## 3. Results

Seven hundred and fifty-two patients with AUGIB symptoms were enrolled in this study (variceal and non-variceal causes of bleeding (Table 1)). Five hundred ninety-two patients (79%) had an endoscopy during the day hours (3:00 *p.m.*–10:00 *p.m.* working hours and 8:00 *a.m.*–10:00 *p.m.* Saturday and Sundays and Bank Holidays) and one hundred and sixty patients (21%) were treated at night hours (10:00 *p.m.*–8:00 *a.m.*) (Table 1). There was a difference in the number of gastroscopies performed during working weekdays (Monday–Friday) and weekends, but this was most likely related to the different duration of the endoscopy team’s duty (16 h vs. 24 h); however, the differences were not statistically significant. The season of the year did not influence the frequency of AUGIB endoscopic interventions.

In the day-hours group patients, the ’pre-endoscopy’ Rockall score assessment was available in 317 cases; 183 of them scored equal to or more than three points and 134 had less than three points. Out of these 134 patients, 63% had active bleeding during the endoscopy (Table 2). In the night-hours group of patients, the ‘pre-endoscopy’ Rockall score assessment was available in 66 cases; 33 of them had less than three points, and among them, 76% had active bleeding during the endoscopy. In the day-hours group, the Glasgow–Blatchford score assessment was possible in 105 patients, and 71% of patients with less than six points had active bleeding (Table 2). In the night-hours group, three out of 19 patients had less than six points, and two of them had active bleeding. In the day-hours group, the mean score for the ‘pre-endoscopy’ Rockall score was three points (IQR 1–5), and that for the Glasgow–Blatchford score was 10 (IQR 6–11). In the night-hours group, the mean score for the ‘pre-endoscopy’ Rockall Score was 2.5 (IQR 1–4), and for the Glasgow–Blatchford score, the mean was 10 (IQR 6–13). 

The overall success rate of therapeutic gastroscopy was 83.5% in the examined population; for the day-hours group, it was 85.2%, and for the night-hours group, it was 77.6% (Table 1). Successful endoscopic treatment was more frequent in patients with non-variceal bleeding (68% vs. 48%), particularly in the subgroup with gastric ulcers (17% vs. 6%). 

**Figure 1:**
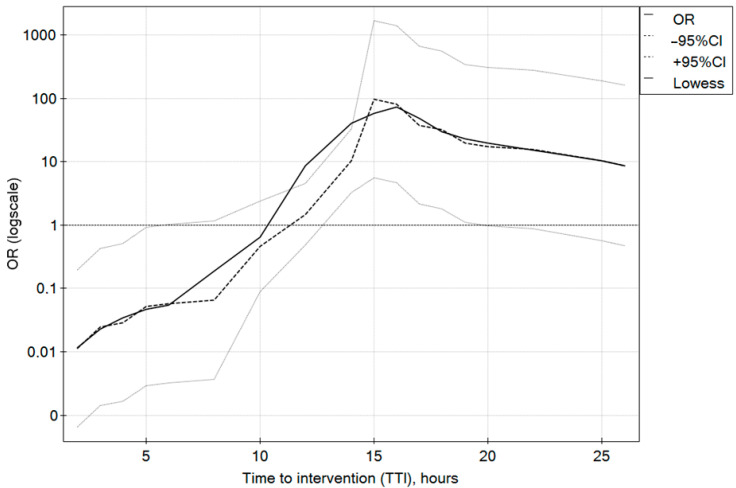
Odds ratio profiles for successful endoscopic intervention in time to intervention (TTI) since the beginning of AUGIB symptoms for the day-hours group.

**Figure 2:**
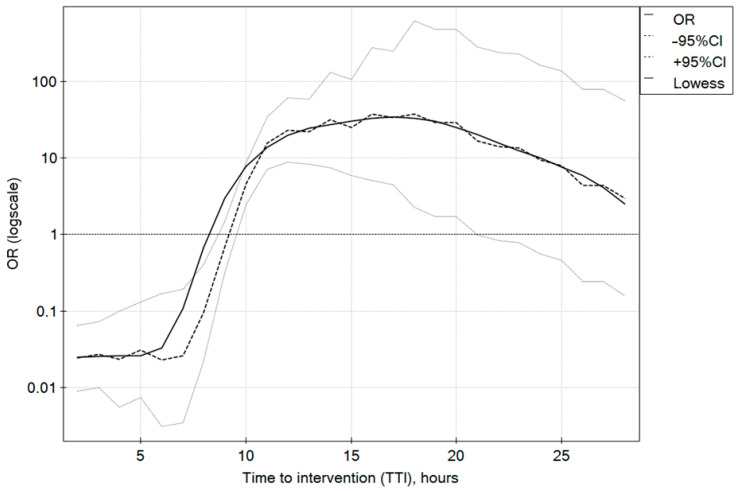
Odds ratio profiles for successful endoscopic intervention in time to intervention (TTI) since the beginning of AUGIB symptoms for the night-hours group.

The mean time from the onset of bleeding symptoms to gastroscopy during nighttime was 6 h (IQR 4–16), which was significantly shorter than in the day-hours group (10 h; IQR 6–15), *p* < 0.001 (Table 1). The time between the onset of symptoms and intervention has a statistically significant effect on the effectiveness of endoscopic bleeding control in all studied groups. In both the day-hours and night-hours groups, a 6 h deferral of intervention from the onset of AUGIB symptoms improved the success rate. In the day-hours group, the effectiveness of endoscopic intervention was at its highest for gastroscopies performed 10 h after the onset of symptoms, and in the night-hours group, this occurred after 8 h (IQR 5–14); the outcomes were poorest up to 5 h in both groups. In both the day-hours and night-hours groups, early endoscopic intervention from the onset of symptoms was associated with a lower chance of success. The time to intervention (TTI) between the eighth and fifteenth hour increased the chances of effectiveness and the cut-off value of the sixth-hour results from the ROC curves. The odds ratio profiles for the successful endoscopic interventions according to the TTI for the day-hours and night-hours groups are shown in Figure 1 and Figure 2, respectively.

A marginal disparity was observed in the frequency of ‘second look’ endoscopies, with a higher occurrence noted during the night hours (*p* < 0.05). In the day-hours cohort, the average intervention time was 23 min, whereas in the night-hours group, it extended to 26 min (*p* < 0.02). The average time required to endoscopically arrest variceal bleeding was 27 min (IQR 15–32), significantly exceeding (*p* < 0.001) the intervention duration for other (non-variceal) causes of bleeding, which averaged at 21 min (IQR 9–27).

**Figure 3:**
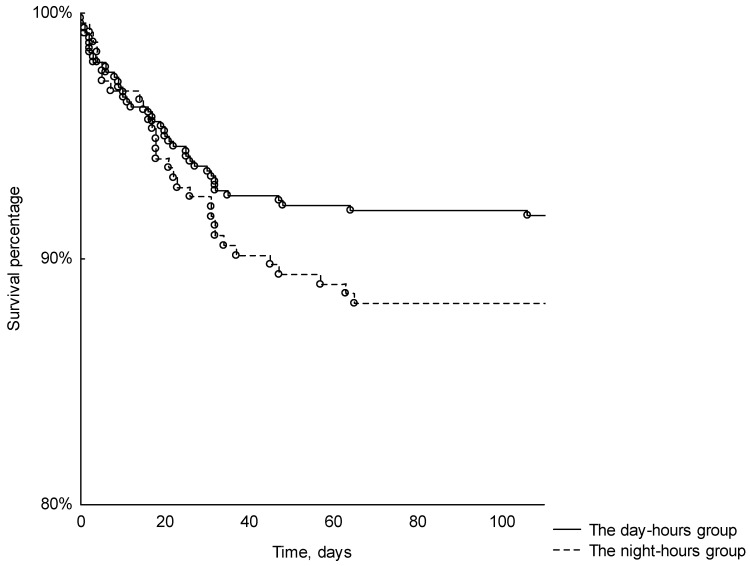
Kaplan–Meier curve for 120-day survival for day-hours and night-hours groups (*p* < 0.29).

Although the night-hours group exhibited a slightly elevated 120-day all-cause mortality compared to the day-hours group, this difference did not attain statistical significance (*p* < 0.26) (Figure 3). The multivariate analysis, encompassing the endpoint of a successful endoscopic hemostasis and significant predictor variables, revealed a superior likelihood of procedural efficacy during the daytime compared to nighttime (OR 3.09, 95% CI 1.5–6.37), as well as a heightened probability of success for non-variceal bleeding (OR 2.29, 95% CI 1.15–4.57). Interestingly, the endoscopist’s experience did not exert a statistically significant impact on procedural effectiveness.

## 4. Discussion 

In the current study, patients were assessed in terms of the effectiveness of an emergency endoscopy for AUGIB, with a particular consideration of the time of day in which the gastroscopy was performed, i.e., whether therapeutic gastroscopy performed between 8:00 *a.m.* and 10:00 *p.m.* is equally effective and safe as that performed between 10:00 *p.m.* and 8:00 *a.m.* Our results indicate a trend towards benefit in the effectiveness of gastroscopy performed before 10 *p.m*. A similar observation was presented in the study by Lau et al., which aimed to determine whether urgent endoscopy for AUGIB in 516 patients with a high risk of death and re-bleeding improves the results [19]. Patients were randomly assigned into two groups: urgent endoscopy (<6 h) or early the next day (<24 h, but >6 h). The authors conducted a post hoc analysis to examine the relationship between death and further bleeding, and the time of the day at which the endoscopy was performed. The highest mortality was observed in a group of patients treated during night hours (between midnight and 5:59 *a.m.*), but the difference was not statistically significant.

In the available literature, there are single studies that analyze the performance of urgent endoscopy due to AUGIB at night, i.e., from 10 *p.m.* to 8 *a.m.* The aforementioned Lau et al. study reported a total of 17 patients who had an endoscopy between midnight and 6 *a.m.*, but this study aimed to analyze the timing from gastroenterology consultation to endoscopy [19]. Hearnshawn et al. presented the results of a nationwide endoscopy audit for acute upper gastrointestinal bleeding (AUGIB) [20]. They reported that 2.8% (142 patients) were examined and treated with endoscopy at night (between midnight and 8 *a.m.*); they also did not refer to the efficacy, but rather to the timing between consultations and endoscopy. 

The present study characterizes the largest population of patients treated for AUGIB in the night hours assessed up to date. Our patients who were treated endoscopically for AUGIB during the night hours had a shorter time to intervention from the onset of symptoms in comparison to the day-hours patients. A reliable identification of the reasons responsible for the shorter interval between symptom onset and endoscopic intervention during the night hours that we note in the analyzed material was not possible; however, organizational factors, like the better availability of anesthesia teams, the quicker arrival of the intervention/endoscopic team to the hospital, fewer patients in the emergency room in the night time, and sometimes, possible inpatients of the physician on duty, might be responsible for the observed effect. 

The meta-analysis performed by Jung et al. based on five retrospective studies presents the impact of the time that has passed from the occurrence of AUGIB symptoms to endoscopy on mortality and re-bleeding rates [13]. The analysis covered 854 patients who received an endoscopy within <12 h and 435 patients who were intervened >12 h after the onset of AUGIB. No clear effect of the time of the endoscopy on the mortality or frequency of re-bleeding was documented. In turn, Huh et al., in a retrospective study covering 411 patients with acute variceal bleeding in the course of cirrhosis, showed worse results for urgent endoscopy (<12 h), especially in patients from the ‘low-risk’ group, which is probably due to the inadequate and insufficient resuscitation and preparation of the patient for therapeutic endoscopy [21]. Similar results were obtained in the presented analysis. In both the variceal and non-variceal bleeding groups studied herein, a shorter interval between the onset of symptoms and intervention reduced the chance of successful treatment. Laursen SB et al. showed that endoscopy performed between the 6th and 24th hour from the onset of AUGIB symptoms is associated with lower mortality compared to patients treated within <6 h or after 24 h from symptom onset, in contrast to Cho et al., who opted for an urgent endoscopy (<6 h) [22,23]. The results of a study by Lau et al., in which patients with AUGIB, mainly of non-variceal etiology, were included, are also in favor of deferred endoscopy, i.e., beyond 6 h from AUGIB symptom onset [19]. In this study, patients received gastroscopies in an urgent or early manner. The analysis of the 30-day mortality and recurrence of bleeding revealed that endoscopy carried out within <6 h, compared to early endoscopy (<24 h), was not superior, especially in patients responding to liquid resuscitation. Similar results have been published in other observation studies, including two systematic reviews [24,25] and three randomized studies [26,27,28]. 

According to the results of Edelson et al. of patients with a suspicion of variceal bleeding, there are no objective criteria for the time that should pass from the first symptoms to endoscopy; the only indisputable prognostic urgency indicators of endoscopic therapy are severe general condition, shock, infection, and hepatocellular carcinoma [29]. The ESGE recommends endoscopy within 24 h in hemodynamically stable patients, and within 12 h in patients with hemodynamic instability which persists despite resuscitation [2,18]. Guo et al., in a retrospective cohort study of 6474 patients, proved that the urgent timing of endoscopy in AUGIB had worse outcomes compared with early endoscopy in terms of the 30-day all-cause mortality, repeated endoscopy rates, and ICU admission rates [30]. In this study, 286 patients who were hospitalized due to variceal bleeding, and 6188 due to non-variceal bleeding, were assessed. Comparing urgent (<6 h), early (6 h–24 h), and late (>24 h) endoscopy, the conclusion was that in this group of patients, especially with uncommon bleeding, early intervention has better results vs. urgent and late, which at the same time emphasizes the importance of the proper preparation and equalization of the patient and the proper implementation of pharmacotherapy and liquid resuscitation. Meanwhile, in this study, deferred endoscopy (24 < t ≤ 48 h post admission) was associated with a higher 30-day mortality and in-hospital mortality as well as 30-day transfusion rates. Our results are in concordance. The effectiveness of the emergency endoscopic intervention, both in the day-hours group and the night-hours group, was comparable if gastroscopy was performed after the sixth hour from the onset of AUGIB symptoms.

The slightly worse outcomes of patients treated at night observed in our study deserve further verification. Our results, if confirmed, can impact the organization of 24 h endoscopic emergency duties in hospitals. The concept of emergency endoscopy care includes the 24 h availability of experienced qualified physicians and nurses, both trained in therapeutic gastrointestinal endoscopy, as well as a qualified anesthesiology team and the appropriate care before and after endoscopy. In light of our results, it may be justified not to undertake endoscopic interventions at night, i.e., between midnight and 8 *a.m*.

## 5. Conclusions

The urgent endoscopic treatment of AUGIB in a round-the-clock duty mode is effective when performed during the daytime as well as at nighttime in a large tertiary hospital. This study showed that the effectiveness of therapeutic gastroscopy in patients with upper gastrointestinal bleeding differs depending on the etiology. The therapeutic endoscopies performed during the night seemed to be slightly less beneficial to patients than those performed during the day. Extending the interval beyond 6 h between symptom onset and therapeutic endoscopy improves treatment outcomes. Appropriate qualifications for the gastroscopy, timing, and risk assessment are of special importance.

## Figures and Tables

**Table 1:** Characteristics of the studied groups.

Variable	All Group	The Day-Hours Group	The Night-Hours Group	*p* Value	95% CI	OR
Sex, Male/Female	480/272	375/214	105/57	<0.79		
Age, median (IQR)	62 (50–74)	62 (50–75)	61.5 (50–72)	<0.38		
Duration time of the gastroscopy, minutes, median (IQR)	18 (10–29)	17 (10–28)	21 (11–34)	<0.01	1.00–1.02	1.01
Variceal etiology of the bleeding, *n* (%)	174 (23.1%)	127 (21.79%)	47 (28.13%)	<0.09		
Esophageal varices, *n* (%)	147 (19.6%)	108 (18.41%)	39 (23.75%)	<0.13		
Gastric varices, *n* (%)	47 (6.2%)	85 (14.53%)	18 (10.63%)	<0.55		
Non-variceal etiology of bleeding, *n* (%): - Duodenal ulcer, *n* (%) - Stomach ulcer, *n* (%) - Sphincterotomy, *n* (%) - Dieulafoy malformation, *n* (%) - Mallory–Weiss syndrome, *n* (%) - Esophageal ulcer, *n* (%) - Surgical anastomosis, *n* (%) - Mucosal bleeding, *n* (%) - Vascular changes, *n* (%) - Cancer, *n* (%) - Fistula, ampullectomy, necrosectomy, *n* (%) - GAVE, *n* (%)	332 (44.2%) 133 (17.7%) 78 (10.4%) 23 (3.1%) 18 (2.4%) 13 (1.7%) 12 (1.6%) 10 (1.3%) 9 (1.2%) 9 (1.2%) 8 (1%) 8 (1%) 3 (0.4%)	267 (45.10%) 109 (18.44%) 60 (10.15%) 15 (2.55%) 15 (2.53%) 12 (2.03%) 8 (1.68%) 5 (1.05%) 5 (1.06%) 4 (0.84%) 5 (1.06%) 4 (0.84%) 2 (0.34%)	65 (40.625%) 24 (15%) 18 (11.32%) 8 (4.91%) 3 (1.88%) 1 (0.63%) 4 (1.43%) 5 (1.80%) 4 (1.43%) 5 (1.80%) 3 (1.08%) 4 (1.44%) 1 (0.62%)	<0.31 <0.32 <0.67 <0.13 <0.85 <0.39 <0.33 <0.03 <0.10 <0.01 <0.28 <0.05 <0.62		
Glasgow–Blatchford score, median (IQR)	10 (6–12)	10 (6–11)	10 (6–13)	<0.48		
‘Pre-Endoscopy’ Rockall score, median (IQR)	3 (1–4)	3 (1–5)	2.5 (1–4)	<0.50		
Endoscopically achieved hemostasis rate/‘success rate’	425/509 (83.5)	334/391 (85)	91/118 (77)	<0.03	0.90–2.32	1.44
Second-look endoscopy, *n* (%)	178 (23.8%)	130 (22.24%)	48 (29.75%)	<0.26		
Time to intervention (TTI), hours (IQR)	9 (5–15)	10 (6–15)	6 (4–16)	<0.001	1.01–1.10	1.10
Time of hospital stay, days (IQR)	10 (5–26)	10 (4–24)	12.5 (5–27)	<0.33		
Mean TTI in ‘successful hemostasis group’, hours (IQR)	10 (7–13)	10 (8–13) *	8 (5–14) **	<0.06	0.97–1.12	1.04
Mean TTI for ‘failed hemostasis group’, hours (IQR)	4 (4–5)	4 (4–5) *	4 (3–4) **	<0.01	0.99–2.49	1.57
Re-bleeding, *n* (%)	131 (17.4%)	98 (16.72%)	33 (20%)	<0.44		
120-day all-cause mortality, *n* (%)	72 (10.6%)	41 (8.65%)	31 (11.15%)	<0.26		

IQR—interquartile range; CI—confidence interval; OR—odds ratio; *—for the day-hours group, the difference between ‘success’ and ‘failure’ endoscopic hemostasis was *p* < 0.00001; **—for the night-hours group, the difference between ‘success’ and ‘failure’ endoscopic hemostasis was *p* < 0.00001; GAVE—gastric antral vascular ectasia.

**Table 2:** Comparison of pre-endoscopic assessment using the Rockall and Glasgow–Blatchford scores for AUGIB between the day-hours and night-hours groups.

	All	The Day-Hours Group	The Night-Hours Group	*p* Value
Endoscopically Confirmed Bleeding (ECB), *n*/N (%),	509/752 (68)	390/590 (66)	119/162 (74)	<0.76
ECB ≥ 3 points in Rockall score, *n*/N (%),where *n* is ECB and N is number of pts with Rockall score ≥ 3	146/216 (68)	120/183 (66)	26/33 (79)	<0.14
ECB < 3 points in Rockall score, *n*/N (%), where *n* is ECB and N is number of pts with Rockall score < 3	109/167 (65)	84/134 (63)	25/33 (76)	<0.16
ECB ≥ 6 points in Glasgow–Blatchford score, *n*/N (%), where *n* is ECB and N is number of pts with GBS ≥ 6	70/103 (68)	56/87 (64)	14/16 (88)	<0.13
ECB < 6 points in Glasgow–Blatchford score, *n*/N (%), where *n* is ECB and N is number of pts with GBS < 6	13/21 (62)	11/18 (61)	2/3 (67)	<0.65

*n*—number of patients with data available, N—entire study group.

## Data Availability

The data presented in this study are available on request from the corresponding author. The data are not publicly available due to privacy issues.
